# Personal

**Published:** 1890-05

**Authors:** 


### Personal.

William C. Krauss, M. D., has removed to 382 Virginia street.

